# Effects of Ca/Si Ratio, Aluminum and Magnesium on the Carbonation Behavior of Calcium Silicate Hydrate

**DOI:** 10.3390/ma12081268

**Published:** 2019-04-18

**Authors:** Jing Li, Qijun Yu, Haoliang Huang, Suhong Yin

**Affiliations:** School of Materials Science and Engineering, South China University of Technology, Guangzhou 510640, China; Jing--Li@hotmail.com (J.L.); concyuq@scut.edu.cn (Q.Y.); h.l.huang@msn.com (H.H.)

**Keywords:** C-S-H, Ca/Si ratio, Al/Si ratio, Mg/Si ratio, carbonation behavior

## Abstract

The effects of Ca/Si ratio, aluminum and magnesium on the carbonation behavior of calcium silicate hydrate (C-S-H) were investigated by using X-ray powder diffraction (XRD), nuclear magnetic resonance (NMR) and thermogravimetric analyzer (TGA). The results showed that the Ca/Si ratio, Al/Si ratio and Mg/Si ratio had a significant influence on the structure, carbonation products and carbonation resistance of C-(M)-(A)-S-H. The mean chain length of silicate chains in C-S-H increased as the Ca/Si ratio decreased. Aluminum uptake in C-S-H increased the content of bridging silicate tetrahedron (Q^2^). A cross-linked structure (Q^3^) appeared when magnesium uptake in C-S-H. The carbonation product of C-S-H was vaterite if the Ca/Si ratio was lower than 0.87. The carbonation products of C-S-H were vaterite and calcite if the Ca/Si ratio was higher than 1.02. C-M-S-H had more polymerized units, stronger bond strength and better carbonation resistance than C-S-H.

## 1. Introduction 

Calcium silicate hydrate (C-S-H) is the main reaction product of alkali-activated slag (AAS) [1]. The carbonation of C-S-H is a process of decalcification when CO_2_ reacts with calcareous phases, resulting in the decomposition of C-S-H, the formation of calcium carbonate with different crystalline forms (i.e., calcite, aragonite and vaterite) and silica gel, and damage to AAS [2,3,4]. Therefore, the carbonation resistance of C-S-H is one of the key factors governing the durability of AAS [5,6]. 

Many researchers studied the carbonation behavior of C-S-H to improve the carbonation resistance of cementitious materials. According to literatures, the carbonation behavior of C-S-H mainly depends on its structure, which is affected by the Ca/Si ratio, uptake of aluminum and magnesium in C-S-H [7,8,9,10].

C-S-H is a kind of amorphous phase with layer structure [11]. An increment of Ca/Si ratio leads to a reduction of the silicate chain length and of the interlayer spacing [12,13]. In the AAS system, aluminum is most likely incorporated in the C-S-H structure due to the high content of aluminum [8,14]. Many structure models of C-S-H have been proposed [15,16,17]. There is a model named the “Cross-linked Substituted Tobermorite Model” (CSTM) that can explain the structure evolution when aluminum uptake in C-S-H [18]. The positions of aluminum in C-S-H have been widely investigated by mean of nuclear magnetic resonance. There are five kinds of positions for aluminum uptake in C-S-H, including two kinds of Al (IV) [19,20], a kind of Al (V) [21,22] and two kinds of Al (VI) [23]. Al (IV) represents the aluminum enters the pairing (Q^2^) or bridging tetrahedra (Q^3^). If Al (IV) occurs in Q^3^ sites (bridging position), cross-linked structure will appear. Al (V) represents the aluminum enters the position of interlayer. Al (VI-1) represents the aluminum that enters the position of interlayer or in the C-S-H structure by replacing calcium [24]. Al (VI-2) stands for the third aluminate hydrate (TAH) which is a precipitate with C-S–H [25]. In a recent study, reference [26] suggested that the aluminum mainly taken up as Al (VI) in TAH when the Ca/Si ratio of C-S-H was high, otherwise, aluminum mainly enters Al (IV) [26]. As for magnesium, early reports believed that magnesium can enter the C-S-H structure by occupying the position of calcium [25,27,28,29]. However, more recently, Bernard et al. studied the effect of magnesium on C-S-H [10,30,31]. They suggested that magnesium will react with silicate ions to form magnesium silicate hydration (M-S-H) [10]. 

The Ca/Si ratio, aluminum and magnesium have effects not only on the structure of C-S-H but also on the carbonation resistance of C-S-H [7,12,13,14]. To figure out the effect of the Ca/Si ratio on the carbonation behavior of C-S-H without the interference of other phases, a few researchers studied the carbonation behavior of pure C-S-H that achieved by mean of chemosynthesis [5,32,33,34,35]. Black et al. studied the carbonation of synthesized C-S-H (initial Ca/Si ratio: 0.4–1.5), and indicated that C-S-H with low Ca/Si ratio had a better carbonation resistance [36,37]. However, Sevelsted et al. believed that the decomposition rate of C-S-H increased with the decrease of Ca/Si ratio [5], which was not consistent with the aforementioned findings. Therefore, further study on the effect of Ca/Si on carbonation of CSH is necessary. Moreover, the changes in carbonation behavior of C-S-H when Al and Mg are taken in by C-S-H were not clear. 

In this study, C-(M)-(A)-S-H with different Ca/Si ratio, Al/Si ratio and Mg/Si ratio were synthesized by means of the chemical precipitation method. The effects of the Ca/Si ratio, aluminum and magnesium on the structure of C-S-H were investigated by means of X-ray powder diffraction (XRD) and ^29^Si nuclear magnetic resonance (NMR). The carbonation products of C-S-H were analyzed by using X-ray powder diffraction. The content of carbonation products of C-S-H were investigated by means of thermogravimetric analyzer (TGA), XRD and the Rietveld method. In this way, insights into the effects of Ca/Si ratio, aluminum and magnesium on the carbonation behavior of C-S-H were provided.

## 2. Experiments

### 2.1. Synthesis of C-(M)-(A)-S-H with Different Ca/Si Ratio, Al/Si Ratio and Mg/Si Ratio

Preparation of C-S-H with different Ca/Si ratio by using chemical precipitation method. The chemical reagents used in the following work were the analytical grade. The C-S-H with initial Ca/Si ratio from 0.67 to 1.6 were prepared by using the chemical precipitation method [13,15]. Calcium nitrate solution (Ca(NO_3_)_2_·4H_2_O, 150 mL) with molarity of 0.067 M, 0.10 M, 0.12 M, 0.14 M and 0.16 M were added drop by drop into sodium silicate solution with molarity of 0.1 M (Na_2_SiO_3_·5H_2_O, 150 mL), respectively. Sodium hydroxide particles (NaOH) were added into sodium silicate solution to keep the pH value of the mixed solution over 13. Ultrapure water was boiled for 10 min to remove the dissolved carbon dioxide (CO_2_). The mixed solution was stirred constantly during the whole process (1 day). The entire synthesizing process was under a N_2_ atmosphere at a room temperature. A white gel was precipitated immediately as the calcium nitrate solution drops to the sodium silicate solution. Separation of gel from the liquid was conducted by using a centrifugal machine (DL-5-B, Anke, Shanghai, China). After that, the gel was washed twice with ultrapure and decarbonized water to remove the sodium and nitrate ions. Finally, the obtained gel was dried at 60 °C for 7 days in a vacuum drying chamber (DZF-6050, Yiheng, Shanghai, China).

Preparation of C-A-S-H with different Al/Si ratio by using chemical precipitation method. The procedures to synthesize C-A-S-H with different Al/Si ratio were the same as those to synthesize C-S-H. A 0.10 M sodium silicate solution (Na_2_SiO_3_·5H_2_O, 100 mL) was used as the source of silicon, a 0.12 M calcium nitrate solution (Ca(NO_3_)_2_·4H_2_O, 100 mL) was used as the source of calcium, and 0.00125 M, 0.0025 M, 0.005 M, 0.01 M, and 0.02 M aluminum nitrate solution (Al(NO_3_)_3_·9H_2_O, 100 mL) were used as the source of silicon aluminum.

Preparation of C-M-S-H with different Mg/Si ratio by using chemical precipitation method. The procedures to synthesize C-M-S-H with a different Mg/Si ratio were the same as those to synthesize C-S-H. A 0.10 M sodium silicate solution (Na_2_SiO_3_·5H_2_O, 100 mL) was used as the source of silicon, a 0.12 M calcium nitrate solution (Ca(NO_3_)_2_·4H_2_O, 100 mL) was used as the source of calcium, and 0.00125 M, 0.0025 M, 0.005 M, 0.01 M, and 0.02 M magnesium nitrate solution (Mg(NO_3_)_2_·6H_2_O, 100 mL) were used as the source of silicon magnesium.

The detailed information of the samples are listed in Table 1. The initial Ca/Si ratio, Al/Si ratio and Mg/Si ratio of C-(M)-(A)-S-H ranged from 0.67 to 1.6, 0.0125 to 0.2, and 0.0125 to 0.2, respectively. But the real Ca/Si ratio of C-S-H ranged from 0.64 to 1.25, which were measured by mean of X-ray fluorescence (XRF, PANalytical, Almelo, Overijssel, The Netherlands). 

### 2.2. Experiment of Carbonation 

All the samples were ground into powder and passed through a sieve with 75 μm before carbonation. Powders of the prepared C-S-H were exposed to atmospheric CO_2_ for 1 day, 7 days, and 28 days with temperature of 20 ± 2 °C and humidity of 65% ± 5%.

### 2.3. Polymerization of Silicon Chain

The data of ^29^Si nuclear magnetic resonance (NMR) were recorded on an AVANCE III 400 NMR spectrometer (Bruker, Fällanden, Zürich, Switzerland). The mean silicate chain length (CL) of C-S-H was calculated as follows [5]:

(1) $$\bar{\mathrm{CL}}\;=\;2(Q^{1}\;+\;{Q_{b}}^{2}\;+\;{Q_{u}}^{2}\;+\;{Q_{P}}^{2})/Q^{1}$$

where Q_b_^2^ is the bridging tetrahedron with calcium; Q_u_^2^ is the bridging tetrahedron with an H^+^; Q_p_^2^ is the pairing tetrahedron. 

### 2.4. The Carbonation Products

The mineralogical identification of C-(M)-(A)-S-H before and after carbonation was analyzed by XRD (PANalytical, Almelo, Overijssel, The Netherlands). XRD patterns were recorded on a Panlytical X’Pert PRO X-ray diffraction with Cu-Kα radiation. The X-ray tube was operated at 40 kV and 40 mA. Values for qualitative analysis were recorded at 2θ intervals from 5° to 70° with a step size of 0.033° for 10 s.

### 2.5. The Content of Carbonation Products

#### 2.5.1. The Content of Carbonation Products for C-S-H 

The quantitative analysis of carbonation products of C-S-H with different Ca/Si ratio was carried out by using a thermogravimetric analyzer (TGA, STA 182 449C, Netzsch, Selb, Bavaria, Germany) with a heating rate of 10 °C per minute in N_2_ atmosphere from room temperature to 1000 °C. Samples were characterized by mean of TGA with and without carbonation. The amounts of CaCO_3_ is determined directly via the weight loss of samples at the temperature of 500–900 °C instead of tangent method [38,39]. The content of carbonation products (CCP) was calculated as follows:

CCP = WL_2_/T_2_ × 100% – WL_1_/T_1_ × 100% (2)

where, CCP is content of carbonation products; WL_1_ is the weight loss of a sample in decomposition peak of CaCO_3_ before carbonation; T_1_ is the total weight of corresponding sample before carbonation in TGA test; WL_2_ is the weight loss of a sample in decomposition peak of CaCO_3_ after carbonation; T_2_ is the total weight of corresponding sample after carbonation in TGA test.

#### 2.5.2. The Content of Carbonation Products for C-A-S-H and C-M-S-H

The quantification of carbonation products of C-A-S-H and C-M-S-H was studied by means of XRD and Rietveld method. Values for quantitative analysis were recorded at 2θ intervals from 10° to 70° with a step size of 0.017° for 20 s The internal standard substance was brucite with content of 15 wt. %.

## 3. Results and Discussion 

### 3.1. The Effects of Ca/Si, Al/Si and Mg/Si Ratios on the Structure of C-(M)-(A)-S-H 

**Figure 1:**
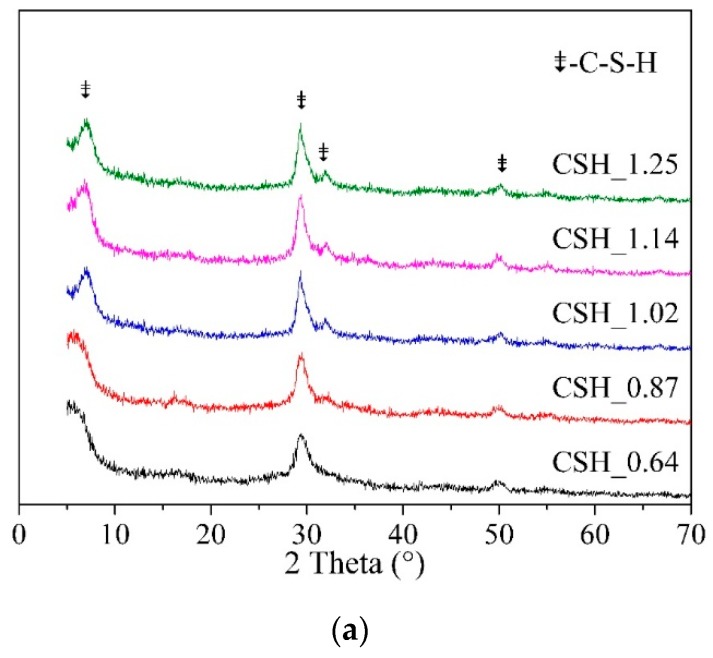

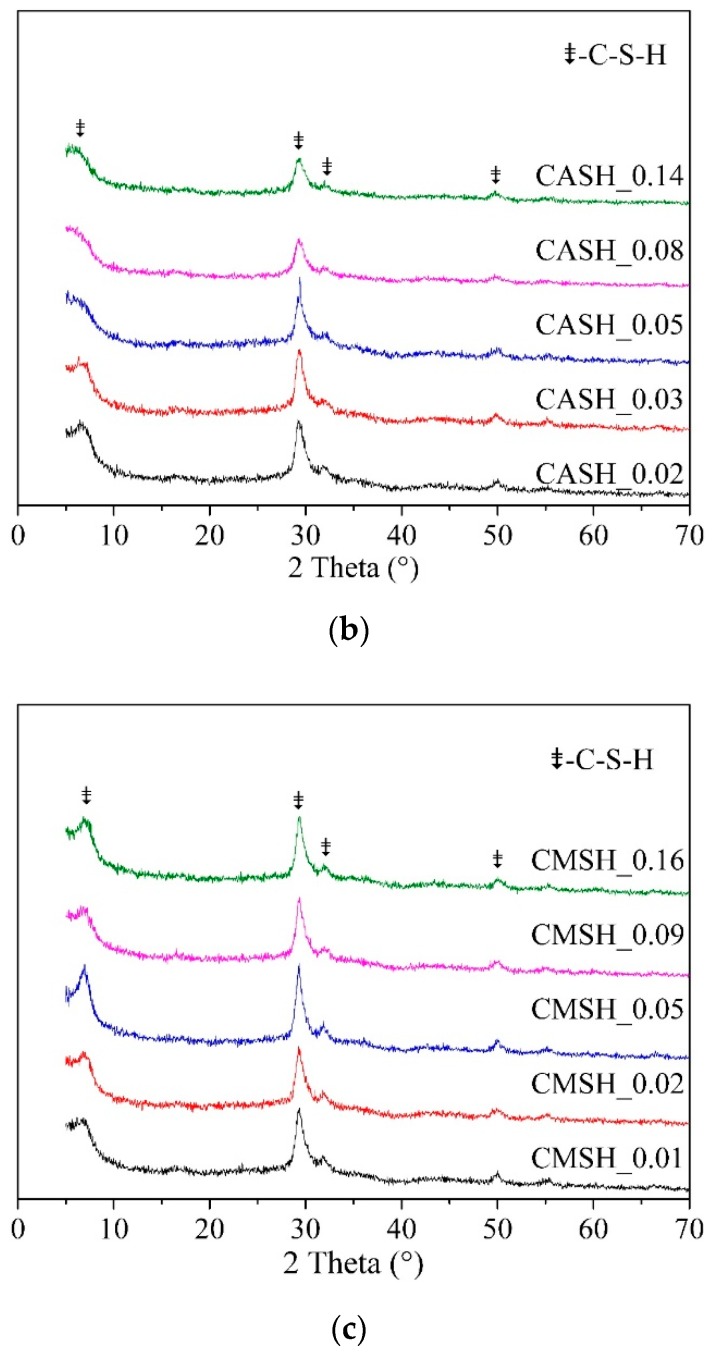
X-ray diffraction (XRD) analysis of synthesized C-(M)-(A)-S-H: (**a**) Ca/Si; (**b**) Al/Si; (**c**) Mg/Si.

The real Ca/Si ratio, Al/Si ratio and Mg/Si ratio of synthesized C-(M)-(A)-S-H were presented in Table 1 after being measured using X-ray fluorescence. The real Ca/Si ratio, Al/Si ratio and Mg/Si ratio of synthesized C-(M)-(A)-S-H were range from 0.64 to 1.25, from 0 to 0.14, and from 0 to 0.16, respectively. It was clear that the real Ca/Si ratio of synthesized C-S-H was smaller than the initial Ca/Si ratio that applied for synthesis, especially for the samples with high initial Ca/Si ratio (Ca/Si >1). The XRD patterns of synthesized C-(M)-(A)-S-H with different Ca/Si ratios, Al/Si ratios and Mg/Si ratios are shown in Figure 1. The characteristic peaks of C-S-H (d: 3.07, 2.80, 1.83; PDF#34-0002) appeared in the all diffraction patterns, indicating the C-S-H could be obtained by means of the chemical precipitation method. 

The effect of Ca/Si ratio on the structure of C-S-H were analyzed by comparing samples CSH_0.64, CSH_0.87, CSH_1.02, CSH_1.14 and CSH_1.25 (see Figure 1a). As the Ca/Si ratio increased, the 2θ value of the small angle diffraction peak (002) increased, which means the distance of the interlayer decreased from about 14 Å to 13 Å. The interlayer distance decreased as the increase of Ca/Si ratio, which was corresponding to the empty bridging silica tetrahedron. The effect of Al/Si ratio on the structure of C-S-H were analyzed by comparing samples CSH_1.02, CASH_0.01, CASH_0.025, CASH_0.05 CASH_0.1 and CASH_0.2 (see Figure 1b). Although they had similar Ca/Si ratios, the 2θ value of small angle diffraction peak of sample C-A-S-H shifted to low angle, indicating that the basal spacing in C-A-S-H was larger than that in C-S-H. It may be because the aluminum entered the structure of C-S-H by substituting silicium that linked across interlayer, which increased the distance of the interlayer. The effect of Mg/Si ratio on the structure of C-S-H were analyzed by comparing samples CSH_1.0, CMSH_0.01, CMSH_0.025, CMSH_0.05 CMSH_0.1 and CMSH_0.2 (see Figure 1c). The presence of magnesium may improve the long-range order of the C-S-H structure, which is known from the increment of diffraction peak intensity. But the d-value of C-M-S-H had no obvious change compared with C-S-H with a same Ca/Si ratio. 

**Figure 2:**
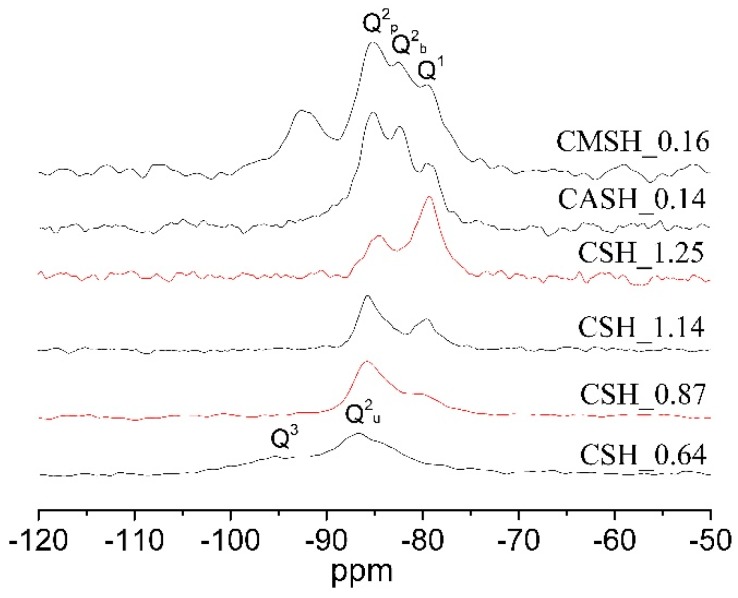
Nuclear magnetic resonance (NMR) spectra of C-(M)-(A)-S-H before carbonation.

To further characterize the structure of C-S-H, parts of samples were then analyzed by using NMR. Figure 2 shows the ^29^Si NMR results of synthesized samples. The Ca/Si ratio, Al/Si ratio and Mg/Si ratio of C-(M)-(A)-S-H had a direct influence on the structure of C-(M)-(A)-S-H. Table 2 gives the corresponding frequencies, band assignments, relative content of Q^n^, and silicate chain length. The main characteristic peaks appeared at around 79 ppm (Q^1^), 83 ppm (Q^2^_b_, bridging position), 85 ppm (Q^2^_p_, pairing position), 88 ppm (Q^2^_u_, bridging position), 95 cm^−1^ (Q^3^), which were typical bands of C-S-H. 

The high content of the Si-O band in the Q^1^ site represented the lower polymerization degree and short silicate chain, and vice versa. As shown in Table 2, the mean chain length of C-S-H increased from 3.11 to 16.77 as the Ca/Si ratio decreased from 1.25 to 0.64, which means C-S-H with a low Ca/Si ratio has a higher degree of polymerization of silicon chain. Moreover, at low Ca/Si ratio (i.e., CSH_0.64), Si-O band in Q^3^ site existed in pure C-S-H. The content of silicate tetrahedron in bridging the position increased when aluminum was added, indicating that part of aluminum enters the structure of C-S-H by replacing the bridge silicium. ^29^Si NMR results also showed that cross-linked silicate groups (Q^3^) existed in sample CMSH_0.16. Reference [40] believed that the Q^3^ site in C-(M)-S-H was the signal for the formation of M-S-H.

### 3.2. The Effect of Time on the Evolution of Carbonation Products of C-S-H with Different Ca/Si Ratio

#### 3.2.1. Carbonation Products

**Figure 3:**
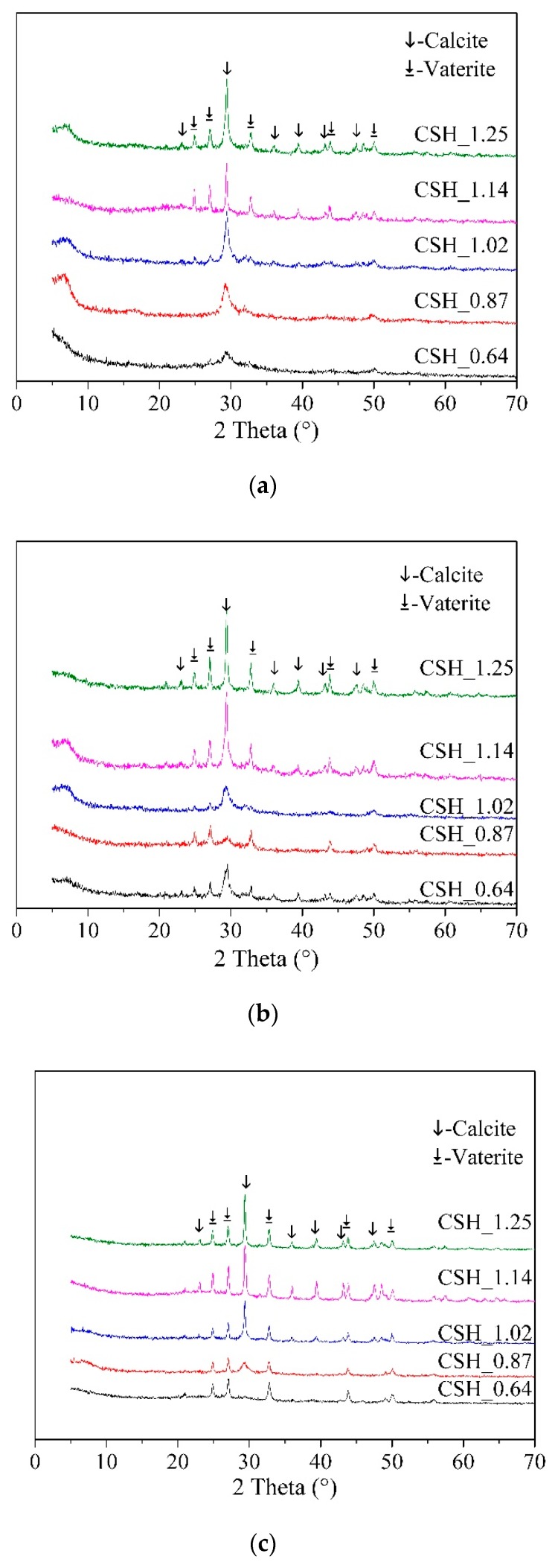
XRD patterns of C-S-H with different Ca/Si ratio after carbonation for (**a**) 1 day, (**b**) 7 days and (**c**) 28 days.

Figure 3 gives the XRD patterns of samples after carbonation for 1day, 7 days and 28 days. Generally speaking, the carbonation products were calcite and vaterite with the same chemical formula expressed as CaCO_3_. The intensity of the diffraction peaks of the carbonation products became stronger as the carbonation time prolonged, indicating the carbonation degree of C-S-H became higher. But the type of carbonation product remain unchanged. At the same carbonation time, C-S-H with a low Ca/Si ratio may have a better carbonation resistance by comparing the intensity of diffraction peaks of CaCO_3_. Besides, in sample CSH_1.02, the formation of calcite was earlier than the formation of vaterite. Therefore, vaterite might not be the transition phase of calcite.

The Ca/Si ratio of C-S-H had a crucial influence on the type of carbonation products. When the Ca/Si ratio of C-S-H was lower than 0.87, i.e., CSH_0.87, the carbonation product was vaterite. When the Ca/Si ratio was higher than 1.02, i.e., CSH_1.02, both vaterite and calcite appeared as carbonation products. It is suggest that C-S-H with low Ca/Si ratio have a calcium octahedral sheets structure, similar symmetries, and a positive charge of the gel surface to vaterite [37]. As for the C-S-H with high Ca/Si ratio, both vaterite and calcite (negative surface charge) are generated at the same time. It implies that Ca-rich C-S-H may include two kinds of structure: one was similar to vaterite, the other was similar to calcite. Therefore, both vaterite and calcite appeared when C-S-H with high Ca/Si ratio carbonated. However, Black et al. believed that vaterite was the main crystal structure as the Ca/Si ration ranged from 0.67 to 1.5 [37]. This result was inconsistent with our experimental result where we demonstrated that the crystal structure of CaCO_3_ varies with the Ca/Si ratio of C-S-H. 

#### 3.2.2. The Effect of Ca/Si Ratio of C-S-H on the Formation Rate of Carbonation Products

**Figure 4:**
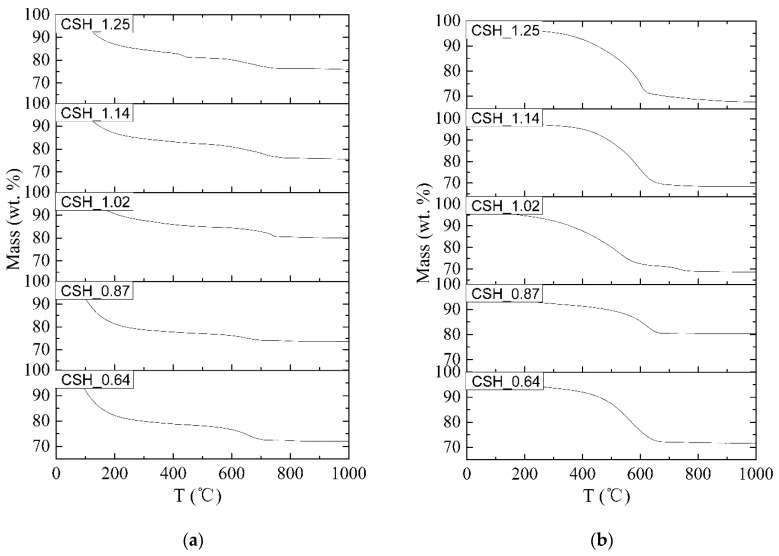
TGA patterns of C-S-H (**a**) before carbonation and (**b**) after carbonation for 28 days.

The content of CaCO_3_ was determined by means of TGA. Figure 4a,b show the TGA patterns of synthesized C-S-H before and after carbonation for 28 days, respectively. The corresponding data of CaCO_3_ content is given in Table 3. The value of CCP corresponding to the content of carbonation products of C-S-H. A small value of CCP represented the low carbonation degree and the strong carbonation resistance of C-S-H. 

According to the results of Figure 4 and Table 3, it is clear that, for all the samples, the content of CO_2_ increased as the carbonation time prolonged. The CCP of C-S-H initially decreased and then increased with the increasing Ca/Si ratio. Sample CSH_0.87 had the smallest value of CCP (6.43), while sample CSH_1.25 had the biggest value of CCP (14.85). The order of carbonation resistance of C-S-H with different Ca/Si ratio was as follows: CSH_0.87 (6.43) > CSH_1.02 (8.70) > CSH_0.64 (13.21) > CSH_1.14 (14.02) > CSH_1.25 (14.85). C-S-H with Ca/Si ratio around 0.9 had a better carbonation resistance which agreed well with the XRD results (Figure 3) and other researchers’ results [37,41]. The CL of C-S-H increased as the decreasing of Ca/Si ratio (Figure 2 and Table 2), implying higher polymerization degree, higher bond strength and shorter bond length. Decalcification of such C-S-H is more difficult because the acting force of (Si-O) to Ca is stronger. This may be the reason why C-S-H with a low Ca/Si ratio has a better carbonation resistant than the one with a high Ca/Si ratio (Figure 4). 

#### 3.2.3. The Structure Change of C-S-H with Different Ca/Si Ratio after Carbonation for 28 Days 

**Figure 5:**
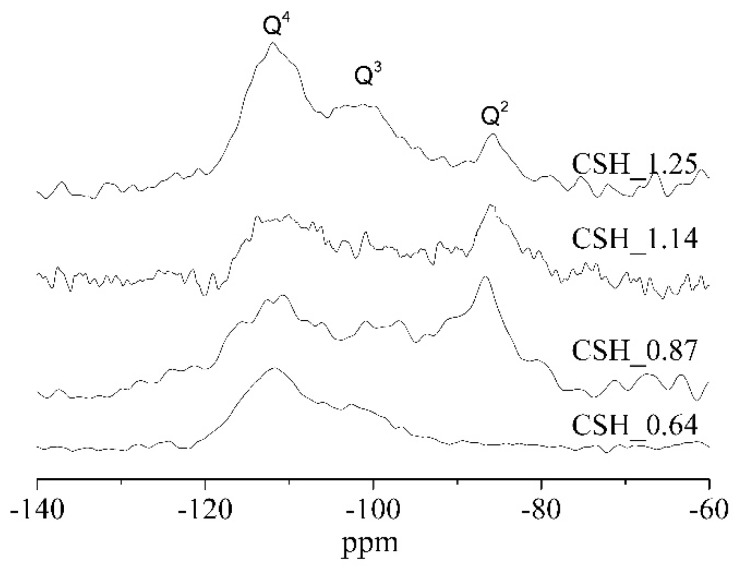
^29^Si NMR spectra of C-S-H after carbonation for 28 days.

Figure 5 shows the ^29^Si NMR spectra of C-S-H after carbonation for 28 days. Combined with the NMR results of C-S-H before carbonation (see Figure 2), it was clear that the intensity of Si-O band in Q^1^ and Q^2^ sites reduced distinctly. Moreover, some new signals were observed at around −112 ppm, −100 ppm in Figure 5, which were assign to Si-O band in Q^4^ and Q^3^ sites. These results indicated that the frequency of Si-O band in C-S-H switched to lower frequency from Q^1^ and Q^2^ sites to Q^3^ and Q^4^ sites, which means the carbonation of C-S-H formed calcium carbonate and silica gel (or C-S-H with low Ca/Si ratio). 

### 3.3. The Effects of Aluminum Uptake and Magnesium Uptake in C-S-H on the Formation Rate of Carbonation Products after Carbonation for 28 Days

#### 3.3.1. The Effect of Aluminum 

**Figure 6:**
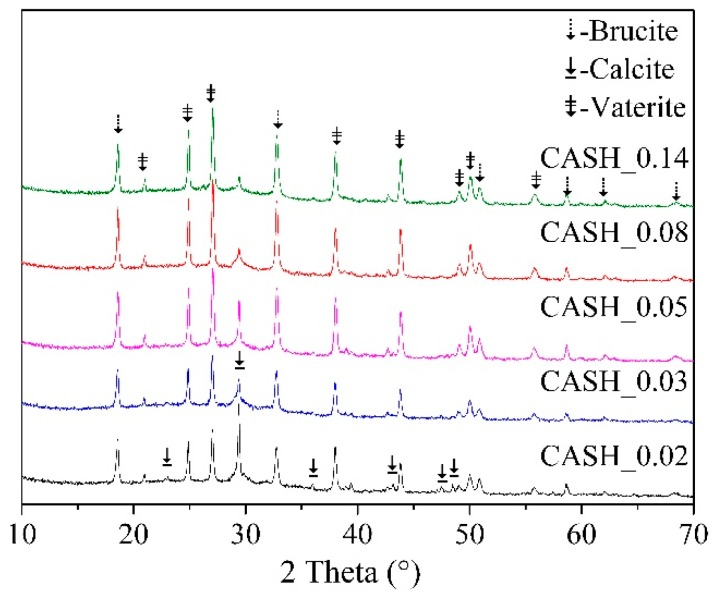
XRD analysis of C-(A)-S-H after carbonation for 28 days.

**Figure 7:**
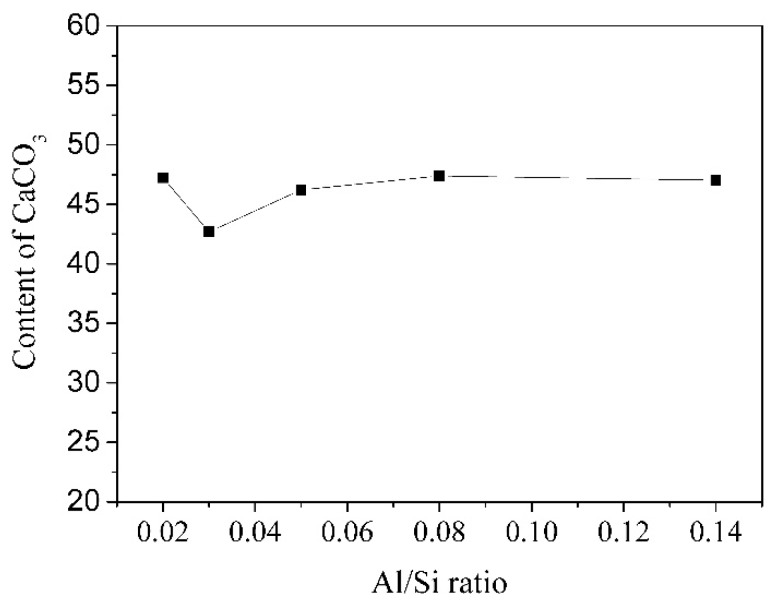
The effect of Al/Si ratio on the content of CaCO_3_.

The effect of Al/Si ratio on the carbonation behavior of C-(A)-S-H was studied by means of XRD and the Rietveld method. The XRD patterns of C-(A)-S-H after carbonation for 28 days are presented in Figure 6. According to Figure 6, the main carbonation products of C-(A)-S-H with Ca/Si ratio around 1.0 were vaterite and calcite. The diffraction peaks’ intensity of calcite became stronger as the Al/Si ratio decreases, indicating the aluminum uptake in C-S-H may hinder to the formation of calcite during the carbonation process. The calculated data of the content of carbonation product is listed in Table 4. It is obvious that the content of calcite was reduced from 13.88 wt. % to 2.77 wt. % as the Al/Si ratio increased. Figure 7 presents the relationship between Al/Si ratio and total content of CaCO_3_. It was clear that no significant changes in the total content of CaCO_3_ as the Al/Si ratio increased, indicating the carbonation resistance of C-S-H was not affected by the aluminum uptake in C-S-H obviously. 

According to Table 2 and Figure 2, the content of Q^2^ in bridging site increased when Al enters the structure of C-S-H, which means C-A-S-H had more polymerized units and stronger bond strength. However, the entrance of aluminum into C-S-H also enlarged the interlayer distance, which increased the porosity of the gel. Therefore, the carbonation resistance of C-S-H has not improved obviously when aluminum entered the structure of C-S-H. 

#### 3.3.2. The Effect of Magnesium 

**Figure 8:**
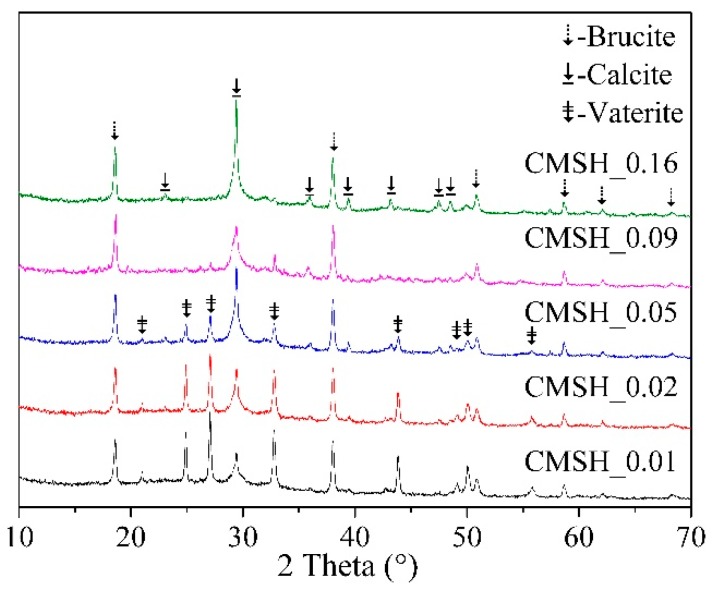
XRD analysis of C-(M)-S-H after carbonation for 28 days.

**Figure 9:**
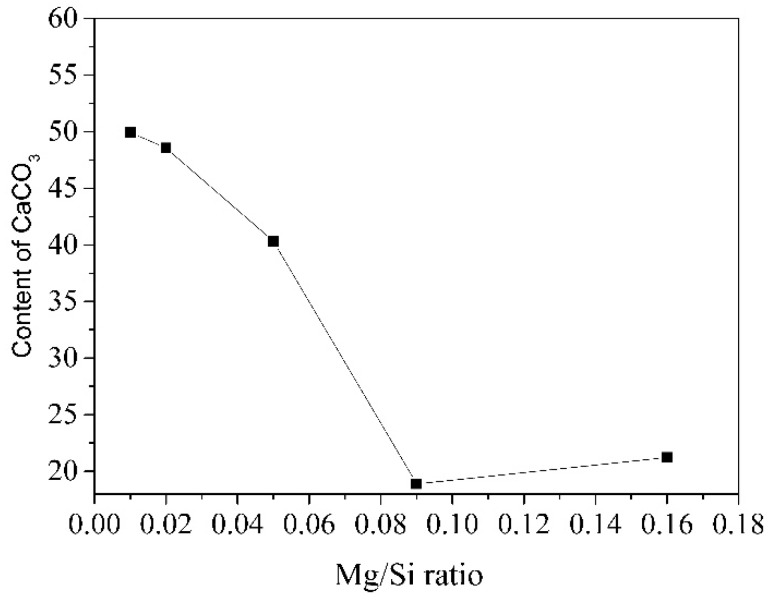
The effect of Mg/Si ratio on the content of CaCO_3_.

The effect Mg/Si ratio on the carbonation behavior of C-(M)-S-H after carbonation for 28 days was also studied by using XRD, as shown in Figure 8. The main carbonation products of C-(M)-S-H were also calcite and vaterite. Unlike Figure 6, the diffraction peak intensity of vaterite became weaker as the Mg/Si ratio increased, indicating the magnesium uptake in C-S-H may hinder the formation of vaterite. The content of calcite and vaterite of carbonated C-(M)-S-H is listed in Table 5. When the Mg/Si ratio increased from 0.01 to 0.16, the content of vaterite decreased from 32.86 wt. % to 1.17 wt. %, while the content of calcite had no obviously change. Figure 9 gives the variation of total content of CaCO_3_ as a function of the Mg/Si ratio of C-(M)-S-H. The total content of CaCO_3_ reduced from 49.94 wt. % to 21.22 wt. % with increasing Mg/Si ratio. The results implied that the magnesium uptake in C-S-H could improve its carbonation resistance, especially when the Mg/Si ratio was bigger than 0.08.

According to Table 2 and Figure 2, a cross-linked structure appeared (Q^3^) when the magnesium uptake in C-S-H occurred, indicating a higher degree of polymerization of silicon chain. Besides, reference [40] believed that the appearance of Q^3^ is the signal of the formation of M-S-H with a clay-like structure, therefore the mixture of C-S-H and M-S-H may be more compact. Both of these two reasons can make the C-(M)-S-H have a better carbonation resistance than C-S-H.

## 4. Conclusions

The effects of Ca/Si ratio, Al/Si ratio and Mg/Si ratio on the structure and carbonation behavior of C-(M)-(A)-S-H were investigated by means of XRD, NMR and TGA. The results showed that the polymerization degree of silicon chain decreased as the Ca/Si ratio of C-S-H increased, which means C-S-H with a low Ca/Si ratio had a higher bond strength and shorter bond length. The content of the Si-O band in bridging site (Q^2^) increased obviously with aluminum uptake in C-S-H, indicating long silicate chains and large interlayer distance. An obvious peak of band Si-O in Q^3^ site appeared with magnesium uptake in C-S-H before carbonation, which was a signal of the formation of M-S-H. Generally speaking, the length of silicate chains in C-(M)-(A)-S-H increased as the Ca/Si ratio decreased and Al/Si ratio and Mg/Si ratio increased.

Due to the differences in the structure of C-S-H, the carbonation products and carbonation resistance were various. For the samples with different Ca/Si ratio, the main carbonation product was vaterite, if the Ca/Si ratio was lower than 0.87. When the Ca/Si ratio was bigger than 1.02, the main products were vaterite and calcite. The C-S-H with a low Ca/Si ratio had a better carbonation resistance. For the samples with a different Al/Si ratio, the carbonation resistance of C-(A)-S-H did not change visibly as the Al/Si ratio increased. Moreover, the content of calcite decreased as the Al/Si ratio increased. For the samples with different Mg/Si ratio, the carbonation resistance of C-(M)-S-H improved obviously, especially when the Mg/Si ratio was bigger than 0.08. Moreover, the content of vaterite decreased as the Al/Si ratio increased. In general, C-(M)-S-H (total CaCO_3_ content of CMSH_0.16 after carbonation for 28 days: 21.22 wt. %) has a better carbonation resistance than C-(A)-S-H (total CaCO_3_ content of CASH_0.16 after carbonation for 28 days: 47.03 wt. %). 

## Figures and Tables

**Table 1:** The detailed information of synthesized samples.

Samples ^a^	Initial Ca/Si Ratio	Initial Al/Si Ratio	Initial Mg/Si Ratio	Real Ca/Si Ratio	Real Al/Si Ratio	Real Mg/Si Ratio	MQA ^b^
CSH_0.64	0.67	0	0	0.64	0	0	TGA ^c^
CSH_0.87	1.0	0	0	0.87	0	0	TGA
CSH_1.02	1.2	0	0	1.02	0	0	TGA
CSH_1.14	1.4	0	0	1.14	0	0	TGA
CSH_1.25	1.6	0	0	1.25	0	0	TGA
CASH_0.02	1.2	0.0125	0	0.77	0.018	0	XRD ^d^
CASH_0.03	1.2	0.025	0	0.80	0.034	0	XRD
CASH_0.05	1.2	0.05	0	0.77	0.054	0	XRD
CASH_0.08	1.2	0.1	0	0.83	0.081	0	XRD
CASH_0.14	1.2	0.2	0	0.86	0.14	0	XRD
CMSH_0.01	1.2	0	0.0125	0.92	0	0.013	XRD
CMSH_0.02	1.2	0	0.025	0.96	0	0.022	XRD
CMSH_0.05	1.2	0	0.05	0.95	0	0.052	XRD
CMSH_0.09	1.2	0	0.1	0.87	0	0.090	XRD
CMSH_0.16	1.2	0	0.2	0.96	0	0.16	XRD

^a^ The initial Ca/Si ratio, initial Al/Si ratio and initial Mg/Si ratio of C-(M)-(A)-S-H were obtained by calculating. The real Ca/Si ratio, real Al/Si ratio and real Mg/Si ratio of C-(M)-(A)-S-H were measured by using X-ray fluorescence (XRF). ^b^ Method for quantitative analysis of the CaCO_3_ content after carbonation experiment. ^c^ TGA is short for thermogravimetric analyzer. ^d^ XRD is short for X-ray diffraction.

**Table 2:** Peak shifts, relative fraction of Q^n^, and mean chain length of C-(M)-(A)-S-H samples.

Samples	Q^1^		Q^2^_p_		Q^2^_b_		Q^2^_u_		Q^3^		CL
	ppm	%	ppm	%	ppm	%	ppm	%	ppm	%	
CSH_0.64	79.00	9.37	84.17	23.16	91.46	9.76	87.00	36.27	95.35	21.44	16.77
CSH_0.87	79.48	24.92	85.75	59329	83.57	15.80	/	/	/	/	8.03
CSH_1.14	79.71	33.47	85.62	41.75	83.77	24.78	/	/	/	/	5.97
CSH_1.25	79.34	64.37	84.54	35.63	/	/	/	/	/	/	3.11
CASH_0.14	79.38	19.53	85.30	44.96	82.40	30.03	88.48	4.28	91.60	1.20	10.12
CMSH_0.16	79.57	20.49	85.43	37.08	82.47	22.31	/	/	92.20	20.08	7.80

**Table 3:** The content of CO_2_ of C -S-H before and after carbonation by mean of thermogravimetric analyzer (TGA).

Samples	Mass Loss (wt. %) from TGA Data ^c^				CCP ^b^
	CO_2__0 ^a^	CO_2__1	CO_2__7	CO_2__28	
CSH_0.64	2.72	4.5	12.37	15.93	13.21
CSH_0.87	2.69	2.97	5.53	9.12	6.43
CSH_1.02	3.87	4.36	5.69	12.57	8.70
CSH_1.14	6.31	9.30	10.60	20.33	14.02
CSH_1.25	4.71	15.95	20.94	19.56	14.85

^a^ CO_2__N: the content of CO_2_ after carbonation for N day, N is the carbonation time. ^b^ CCP: the content of carbonation products = CO_2__28–CO_2__0. ^c^ TGA patterns of C-S-H after carbonation for 1 day and 7 days have not presented in this paper, only TGA data listed in Table 3.

**Table 4:** The effect of Al/Si ratio on the content of calcite and vaterite.

Samples	Calcite	Vaterite	Total CaCO_3_	Amorphous Phase
CASH_0.02	13.88	33.36	47.24	52.76
CASH_0.03	4.90	37.79	42.69	57.31
CASH_0.05	6.92	39.31	46.22	53.78
CASH_0.08	4.18	43.21	47.39	52.61
CASH_0.14	2.77	44.25	47.03	52.97

**Table 5:** The effect of Al/Si ratio on the content of calcite and vaterite.

Samples	Calcite	Vaterite	Total CaCO_3_	Other Phase
CMSH_0.01	17.08	32.86	49.94	50.06
CMSH_0.02	15.19	33.37	48.56	51.44
CMSH_0.05	20.37	19.98	40.35	59.65
CMSH_0.09	16.45	2.45	18.90	81.10
CMSH_0.16	20.05	1.17	21.22	78.78
